# Meteorological Table

**Published:** 1812-02

**Authors:** 


### METEOROLOGICAL TABLE.

*From December* 26, 1811, *to January* 26, 1812.

| | D | Therm. | | | Barom. | | Hygrom. *Dry.——Damp.* | | | Winds. | Atmos. Variation. |
|---|---|---|---|---|---|---|---|---|---|---|---|
| | 27 | 29 | 33 | 32 | $29^{4}$ | 29 | 18 | 22 | 21 | SW. | Fog .. S .. F .... |
| | 28 | 33 | 35 | — | $29^{1}$ | $^{4}$ | 24 | 20 | 23 | N. | S . F .. S ... C .. |
| ○ | 29 | 33 | 34 | 32 | $29^{6}$ | $^{8}$ | 22 | 20 | 25 | N. | C ... — ... — ... |
| | 30 | 29 | 33 | 31 | $29^{9}$ | 30 | 25 | — | — | N. | F ... C .. — .. |
| | 31 | 29 | 32 | 35 | $30^{1}$ | $29^{9}$ | 25 | 20 | — | W. | C .... — .... R .. in N |
| | 1 | 37 | 42 | 38 | $29^{8}$ | $^{7}$ | 26 | 24 | 30 | W.. | F.. — ... — .. |
| | 2 | 40 | 46 | 42 | $29^{6}$ | $^{5}$ | 26 | — | 30 | SW.. | F . — ... R . F .. |
| | 3 | 38 | 43 | 36 | $29^{4}$ | — | 25 | 23 | 25 | SW.. | C ... R .. F ... |
| | 4 | 35 | 40 | 36 | $29^{5}$ | $^{3}$ | 25 | 26 | 27 | SW.·E. | F .... C ... S .. |
| | 5 | 36 | — | 38 | $29^{3}$ | $^{4}$ | 27 | — | 30 | NE.. | S. — ... R .. |
| ◑ | 6 | 33 | 38 | — | $29^{7}$ | — | 28 | 22 | 25 | W.. | F...C..R.F...S...inN |
| | 7 | 34 | 37 | — | $29^{6}$ | $^{7}$ | 25 | — | — | NW.. | C ... S ... F .. |
| | 8 | 36 | 39 | 36 | 30 | $30^{2}$ | 25 | 22 | 25 | NE... | F .. C ... — .. |
| | 9 | 34 | 37 | 35 | $30^{2}$ | $^{1}$ | 26 | 24 | 23 | NW. | F . C ... S ... — ... |
| | 10 | 35 | 37 | 36 | 30 | $^{2}$ | 27 | — | 26 | NW. | C .... — .... — .... |
| | 11 | 37 | 38 | — | $30^{1}$ | $29^{8}$ | 29 | 25 | 28 | W. | C .... — .... — .... |
| | 12 | 37 | 40 | 38 | $29^{8}$ | — | 25 | — | 27 | NE.. | F ... — ... C ... |
| | 13 | 36 | 40 | 39 | $27^{7}$ | $^{8}$ | 25 | 22 | 27 | N. | C ... C ... R. |
| ● | 14 | 39 | 41 | 39 | 30 | $^{1}$ | 25 | 24 | 25 | NW. | C .... F .. C ... |
| | 15 | 38 | 43 | 37 | $30^{1}$ | — | 24 | — | 27 | NW. | F .. — .... R . |
| | 16 | 36 | 40 | — | $30^{1}$ | — | 27 | 24 | 25 | W.. | C ... F .. Fog .. F ... |
| | 17 | 39 | 40 | 39 | $30^{2}$ | — | 26 | — | 24 | NW.. | Fog .... C.... R. in N |
| | 18 | 37 | 43 | — | $30^{2}$ | — | 20 | 25 | 27 | NW. | C .... — ... — ... |
| | 19 | 43 | 46 | 45 | $30^{1}$ | 30 | 28 | — | 26 | W.. | R .. C ... R .. |
| | 20 | 40 | 41 | 37 | $29^{9}$ | $^{8}$ | 25 | 15 | 24 | N.. | F .. — ... — .... |
| ◐ | 21 | 34 | 38 | 33 | $29^{8}$ | — | 19 | 14 | 18 | N.. | F ... — .... — .... |
| | 22 | 35 | 39 | 37 | $29^{8}$ | — | 16 | — | 20 | N.. | C .... F ... R . S . |
| | 23 | 36 | — | 35 | $29^{8}$ | $30^{1}$ | 15 | 11 | 9 | E.. | C .... S . F ... |
| | 24 | 35 | 38 | 35 | $30^{1}$ | — | 13 | 9 | 15 | N. | C .... F .... — .... |
| | 25 | 32 | 41 | 42 | 30 | — | 15 | 13 | 21 | W.. | C .... C ... R . |
| | 26 | 42 | 45 | 40 | $29^{9}$ | 30 | 25 | 20 | — | SW | R .. C ... F .... |

Quantity of rain from December 26 to January 26, 1 inch $\frac{43}{100}$.

4th. The first snow in the new year fell this evening.

5th. Snow great part of the day, but partially dissolving, and changing to rain in the evening.

6th. After rain in the evening, a considerable quantity of snow fell, covering the streets and houses on the morning of the 7th more completely than had before happened this winter. Stormy wind from N. E. in the night.

10th. Dense fog the whole day, occasioning a degree of darkness in the metropolis, greater than had occurred for many years. Shops and public offices were lighted up during the whole day, as in winter evenings. For the greater part of the day it was impossible to read or write at a window without candles. In the corn-market, and some other places of public resort, no business could be done. Instead of the usual character of January, bright and frosty, heavy fogs and clouds have prevailed: the clear days have had the character of April, humid, genial, and warm.

The 12th and 15th were of this kind; and the 16th, though mild, was obscured at times by passing masses of dense fog.

The

The 17th was so dark at nine in the morning as to require candles.

The 25th affords an instance of the little dependance to be placed on personal feelings for ascertaining the actual degree of cold. When the thermometer stood at 41°, the sensation of cold in the open air was painful in the middle of the day, which gave place to a genial softness in the evening, as the breeze from the west prevailed. Extensive Halo.

The lowest point of the thermometer in this interval 29°, the highest 46°. The barometer has had a fluctuation from $29^3$ to $30^2$. The hygrometer has not indicated much humidity, and the degree which did exist has not been liable to considerable variations: in the middle of the 24th day the approach was nearest to the dry side of the scale. The prevailing wind has been from the northern quarter. Eighteen days it has blown from the N., NE., or NW.

